# Malaysia healthcare system: Protect the public or the industry?

**DOI:** 10.34172/hpp.2021.16

**Published:** 2021-05-19

**Authors:** Amirul Ashraf, Siew Chin Ong

**Affiliations:** School of Pharmaceutical Sciences, Universiti Sains Malaysia, Malaysia

## Dear Editor,

Rising healthcare cost has been a dilemma globally. In Malaysia, the out-of-pocket (OOP) expenditure for year 1997-2017, is between 29% to 38% of total health expenditure^1^ - beyond the suggestion of World Health Organization (WHO), of 15%-20%.^2^

Malaysian has always believed that healthcare is a right and it is the responsibility of the government to provide a good quality healthcare for the people. However recently, the government has seems to appease to the private sector rather than protecting the public interest. This can be seen in several recent incidents: the deregulation of the consultation fees, the rejection of separation between prescribing and dispensing, the objection on mandatory prescription upon patient’s request and the latest, which is the rejection of drug price control.

### The rejection of separation between prescribing and dispensing

The separation between prescribing and dispensing has been a conundrum in Malaysia with pharmacist groups continue to push for the reform whereas the doctors groups continue to reject the agenda. The pharmacists argue that the separation will improve patient’s care, avoid potential conflict interest by dispensing doctor and help to reduce medication error. Meanwhile, doctors group argue that the move will increase consultation cost, inconvenience for patients and there is lack of pharmacies in the rural area.^3^

The practice is also recommended by WHO^4^ and is common in developed countries. Thus, Ministry of Health (MOH) should push forward the agenda and do the necessary to make it happen. However instead of helping to reduce patients OOP expenditure, MOH is dragging their feet regarding the matter. Although there are fewer pharmacies in rural areas, the reform can be done in phases, targeting urban areas first, and subsequently extend to more rural areas as the number of pharmacies increased.

### The objection on mandatory prescription upon patient’s request

The government has tabled an amendment to the Poison Act 1952 which obliged the doctors to provide prescription upon request. Violation will result in fine and imprisonment.^5^ The pharmacist group support mandatory prescription for patients as it can help the patients to get their medicine from the pharmacies. The doctors group opposed the amendment saying that it will lead to potential abuse by the patients, although the real concern is that, the mandatory prescription will reduce private clinics’ revenue as patient will buy the medicines from the pharmacies.

### The deregulation of the consultation fees

The doctors’ consultation fee has remained the same since 1990s. The doctors group has repeatedly asked for the government to harmonize the fees with the private sector counterpart. However instead of harmonizing the fees with the private sector, the government has opted to remove the price cap totally.^6^ This spark protest from the consumer groups as it may lead to higher cost and cartel practice.^7^ Instead the group suggested that the fees should be capped according to the inflation rate. Rather than deregulating the fees, the government should keep the status quo and reduce the consultation fees in the private sector. OOP expenditure is already high and fee deregulation will worsen it. It has been shown that high consultation fees deter patients from seeking treatment^8,9^ and promote social inequality.^10^

### The rejection of drug price control

The Cabinet has approved for drug price control in May 2019 which will be implemented in phases, starting with the public sector and gradually extend to the private sector.^11^ The drug price in the private sector is unregulated and left entirely to the market forces. The effort receives strong support from the public and the consumer groups. However, the private sector strongly opposed to it. Recently MOH has backtracked from the regulation saying that it is merely a pilot project and will not affect the pharmaceutical industry.

It is the government’s responsibility to protect and preserve the health of its citizen. However, Malaysia’s recent revision on health issues seems to favour the private sector instead of the health and welfare of its citizens. The policymakers must realize that free market does not exist in the healthcare sector and government intervention is needed. The government must fill the gaps and regulate the market where unfairness occurs and ensure that a high quality healthcare system remain accessible to all Malaysians.

## Acknowledgements

The authors appreciate the reviewers for their insightful comments.

## Funding

No funding received.

## Competing interests

None.

## Ethical approval

This study does not require any approval from the responsible ethics committee as it used secondary data.

## Authors’ contributions

AA collected the data, performed analysis and wrote the manuscript. SCO supervised the project.
